# Tumor suppressive effect of PARP1 and FOXO3A in gastric cancers and its clinical implications

**DOI:** 10.18632/oncotarget.6264

**Published:** 2015-11-02

**Authors:** See-Hyoung Park, Kyu Yun Jang, Min Jae Kim, Sarah Yoon, Yuna Jo, So Mee Kwon, Kyoung Min Kim, Keun Sang Kwon, Chan Young Kim, Hyun Goo Woo

**Affiliations:** ^1^ Department of Physiology, Ajou University School of Medicine, Suwon, Republic of Korea; ^2^ Program in Nano Science and Technology, Department of Transdisciplinary Studies, Seoul National University Graduate School of Convergence Science and Technology, Suwon, Republic of Korea; ^3^ Department of Biomedical Sciences, Graduate School, Ajou University, Suwon, Republic of Korea; ^4^ Department of Pathology, Chonbuk National University Medical School, Research Institute of Clinical Medicine of Chonbuk National University-Biomedical Research Institute of Chonbuk National University Hospital and Research Institute for Endocrine Sciences, Jeonju, Republic of Korea; ^5^ Department of Preventive Medicine, Chonbuk National University Medical School, Jeonju, Republic of Korea; ^6^ Department of Surgery, Chonbuk National University Medical School, Jeonju, Republic of Korea

**Keywords:** olaparib, PARP1, FOXO3A, prognosis, G2/M arrest

## Abstract

Poly (ADP-ribose) polymerase1 (PARP1) has been reported as a possible target for chemotherapy in many cancer types. However, its action mechanisms and clinical implications for gastric cancer survival are not yet fully understood. Here, we investigated the effect of PARP1 inhibition in the growth of gastric cancer cells. PARP1 inhibition by Olaparib or PARP1 siRNA could significantly attenuate growth and colony formation of gastric cancer cells, and which were mediated through induction of G2/M cell cycle arrest but not apoptosis. FOXO3A expression was induced by PARP1 inhibition, suggesting that FOXO3A might be one of downstream target of the PARP1 effect on gastric cancer cell growth. In addition, by performing tissue microarrays on the 166 cases of gastric cancer patients, we could observe that the expression status of PARP1 and FOXO3A were significantly associated with overall survival (OS) and relapse-free survival (RFS). Strikingly, combined expression status of PARP1 and FOXO3A showed better prediction for patient's clinical outcomes. The patient group with PARP1+/FOXO3A− expression had the worst prognosis while the patient group with PARP1−/FOXO3A+ had the most favorable prognosis (OS: *P* = 6.0 × 10^−9^, RFS: *P* = 2.2 × 10^−8^). In conclusion, we suggest that PARP1 and FOXO3A play critical roles in gastric cancer progression, and might have therapeutic and/or diagnostic potential in clinic.

## INTRODUCTION

Gastric cancer is one of the most common malignancies with heterogeneous clinical outcome [1], but its mechanisms leading to development and/or progression of tumors remain unclear. PARP1 is a kind of polymerase that can conjugate ADP from NAD+ to target proteins such as histones and p53, to activate their functions in DNA repair response to DNA damage. PARP1 has been known to play an important role in tumor development in breast, ovary, and skin. Moreover, in most of breast and ovarian cancer patients (about 80%), BRCA gene, another DNA repair gene, is frequently mutated [2]. As a consequence, the expression of PARP1 is up-regulated to compensate the impaired DNA repair and the tumor cells can survive and progress despite of their presence of DNA damage [2–4]. Therefore, PARP1 is thought of as one of therapeutic targets for the development of anti-cancer treatments particularly for BRCA-mutated tumors, and a variety of clinical trials are actively in progress [5–11]. However, recent studies have shown that the expression of PARP1 protein can predict poorer prognosis regardless of the existence of a BRCA mutations [12–16], although its action mechanisms were not fully established. Recently, PARP1 inhibition has been addressed to attenuate the AKT-associated phosphorylation of forkhead box O (FOXO) transcription factors [17, 18]. Of these FOXO transcription factors, FOXO3A has been known as a downstream target of serine/threonine protein kinase B (PKB)/AKT. Phosphorylated FOXO3A by AKT interacted with 14–3–3, resulting in faster degradation of FOXO3A protein [19, 20]. When FOXO3A is activated by inhibition of PI3K/AKT pathway, FOXO3A can promote a wide range of cellular effects including cell cycle arrest, induction of autophagy, sensitization to chemotherapeutics, inhibition of metastasis and cell differentiation, and apoptotic cell death [21, 22]. Indeed, decreased expression of FOXO3A protein was associated with tumor progression in various malignancies [23, 24]. Moreover, clinically used drugs like paclitaxel, imatinib, and doxorubicin have shown therapeutic effects through activation of FOXO3A and its targets [25]. Collectively, PARP1 has been implicated in AKT activity, and the FOXO3A phosphorylation by AKT causes its nuclear exclusion and degradation, resulting in the suppression of its transcriptional activity. Therefore, we hypothesized that PARP1 and FOXO3A may interact together and play critical roles in cancer progression. Moreover, to our knowledge, the functional and clinical roles of PARP1 in gastric cancer were not evaluated vigorously yet.

With respect to these, in the present study, we aimed to investigate whether the expressions of PARP1 and FOXO3A have functional and clinical significance in gastric cancer. By performing cell culture experiments, we could observe that PARP1 inhibition significantly attenuated gastric cancer cell growth, and which were mediated through FOXO3A expression. Furthermore, by performing tissue microarrays on the 166 cases of gastric cancer patients, we demonstrated the prognostic predictability of the expression status of PARP1 and FOXO3A in gastric cancer. Our results may provide new biological and clinical insights on the expressions of PARP1 and FOXO3A in gastric cancer progression.

## RESULTS

### PARP1 inhibition can suppress the growth of gastric cancer cells

**Figure 1:**
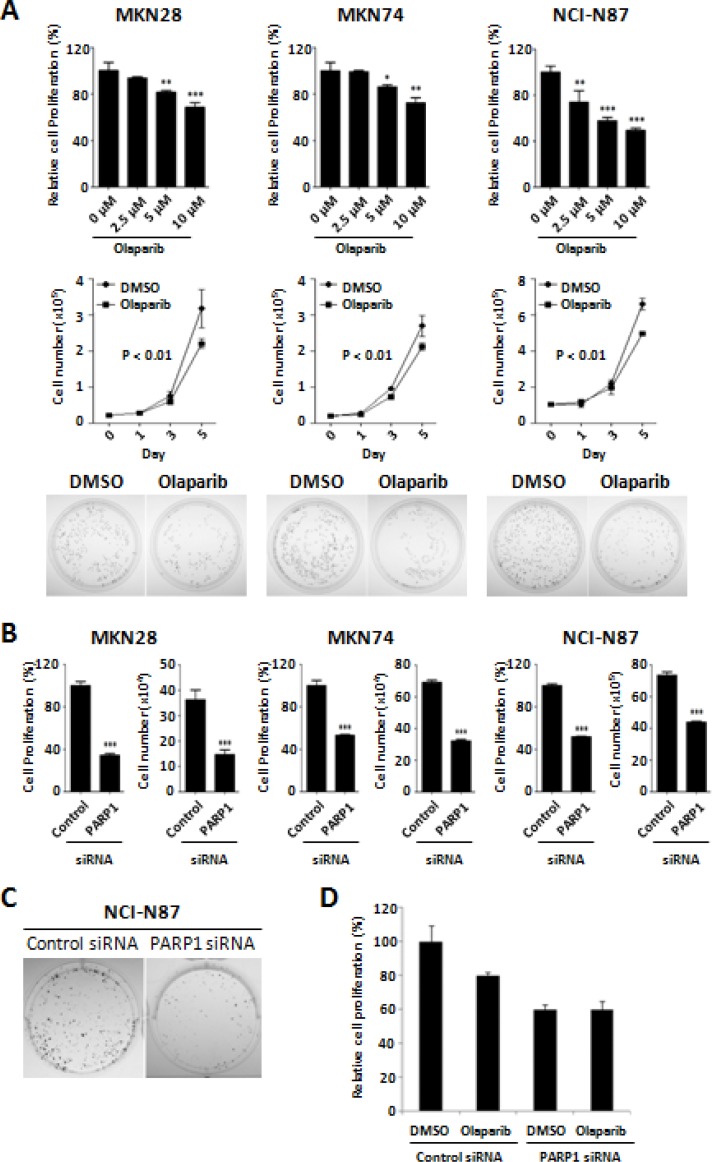
Anti-proliferative activity of Olaparib and PARP1 siRNA against gastric cancer cells (**A**) Dose-dependent effect of Olaparib (0, 2.5, 5, and 10 μM) after 72 h incubation are shown in MKN28, MKN74, and NCI-N87 cells. Relative cell proliferation rate by Olaparib compared to the control (DMSO) treatment was determined by an MTT assay (*top*). Cell counting assays show the time- dependent effect of Olaparib (0, 2.5, and 5 μM) for 0, 1, 3, or 5 days (*middle*). Colony formation assays are performed on the cells treated with Olaparib (0 and 2.5 μM) for 14 days as described in the “Materials and Methods” (*bottom*). (**B**) Cells are transfected with control or PARP1 siRNA (30 nM) for 4 days and cell proliferation is determined by a cell counting assay and an MTT assay. (**C**) Colony formation assays are performed using NCI-N87 cells transfected with control or PARP1 siRNA (100 nM) for 14 days. (**D**) Olaparib (5 μM, for 72 h) is treated on the MKN28 cells transfected with control or PARP1 siRNA, and the cell viability is determined by MTT assay. Results shown are from one representative assay out of three biological replicates. Data are the mean ± S.D. (*n* = 3). *P < 0.05, **P < 0.01 with respective control.

Tumor-suppressive activity of the PARP inhibitor, Olaparib, was evaluated by using different methods of MTT assay, cell counting, and colony formation assays in the three human gastric cancer cell lines of MKN28, MKN74, and NCI-N87. Treatment of Olaparib showed significant suppression of cancer cell growth in a dose- dependent manner (Figure 1A, top). After 72 h incubation, IC50 of Olaparib was approximately 10 μM in the three different cell lines. Cell counting assays confirmed the time-dependent growth inhibition by Olaparib treatment. The cell numbers after treatment with Olaparib (2.5 μM) for 5 days were significantly lower than those of the control cells (Figure 1A, middle). Colony formation ability was also decreased by Olaparib treatment (Figure 1A, bottom). In addition, to confirm the Olaparib effect through PARP1 inhibition, we performed siRNA-mediated knock-down experiments. Similar to Olaparib, knock-down of PARP1 by siRNA significantly inhibited the proliferation and colony formation of gastric cancer cells (Figure 1B and 1C). However, treatment of Olaparib in the PARP1 siRNA-transfected cells had no effect on cell proliferation, which may indicate that the tumor-suppressive effect of Olaparib is through inactivation of PARP1 (Figure 1D). Supporting this, a previous study has demonstrated that PARP1/2 can be trapped into a specific DNA sites causing direct cytotoxicity, suggesting that PARP1 is essential in Olaparib-mediated tumor suppression [26]. Therefore, we suggest that PARP1 inhibition by Olaparib can suppress the growth of human gastric cancer cells.

### PARP1 inhibition induce FOXO3A expression and G2/M cell cycle arrest

**Figure 2:**
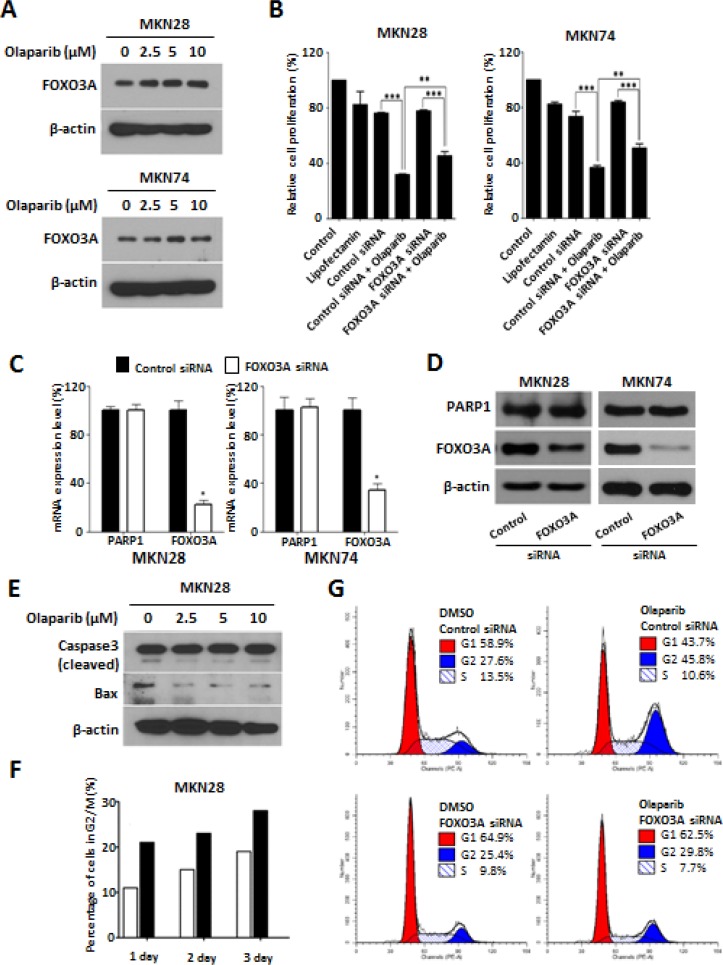
PARP1 inhibition induce G2/M cell cycle arrest and FOXO3A expression (**A**) Western blotting results of cells treated with Olaparib (0, 2.5, 5, and 10 μM) for 72 h. β-actin is used as a gel-loading control. (**B**) Olaparib (10 μM) or control vehicle (DMSO) are treated for 72 h in the MKN28 and MKN74 cells transfected with non-target control or FOXO3A siRNA (30 nM), and the effect on cell proliferation is determined by an MTT assay. (**C**) The expressions of PARP1 and FOXO3A mRNAs are measured by real-time qPCR in the MKN28 and MKN74 cells transfected with non-target control or FOXO3A siRNAs (30 nM for 24 h). Data are the mean ± S.D. (*n* = 3). *P < 0.05 with respective control. (**D**) The expressions of PARP1 and FOXO3A proteins are measured by western blot analysis in the MKN28 and MKN74 cells transfected with non-target control or FOXO3A siRNAs (30 nM for 72 h). (**E**) Western blotting results of cleaved Caspase 3 and Bax expression in the MKN28 cells treated with Olaparib (0, 2.5, 5, or 10 μM) for 3 days. (**F**) Flow cytometry results of MKN28 cells treated with Olaparib (0 or 10 μM) for 1, 2, or 3 days. (**G**) Olaparib (10 μM) is treated for 48 h on the MKN28 cells transfected with control or FOXO3A siRNA. Distribution of cell cycle is analyzed using flow cytometry. These results are from one representative assay of three biological replicates. Data are mean ± S.D. (*n* = 3). **P < 0.05, ***P < 0.01 with respective control.

As described in the Introduction, FOXO3A is thought as one of putative effector downstream target of PARP1. To evaluate this hypothesis, we examined the effect of Olaparib on the expression of FOXO3A. Western blot analysis demonstrated that the treatment of Olaparib up-regulate FOXO3A expression in both MKN28 and MKN74 cells in a dose-dependent manner (Figure 2A). In addition, when Olaparib was treated to the FOXO3A knock-down cells, the Olaparib–mediated growth inhibition was rescued, in part, by knock-down of FOXO3A expression (Figure 2B). By contrast, knock-down of FOXO3A had no effect on the expression levels of PARP1 mRNAs as well as proteins (Figure 2C, 2D). These results consistently support that FOXO3A is one of downstream target for the tumor-suppressive effect of PARP1 inhibitor. Taken together, we suggest that tumor-suppressive effect of PARP1 inhibition is mediated, at least in part, by FOXO3A activation, although further studies might be required to address direct signaling mechanisms between PARP1 and FOXO3A.

FOXO3A has been known to harbor multifaceted cell functions including cell cycle regulation, apoptosis, autophagy, and DNA repair [18, 27]. With this concern, we next examined whether the effect of PARP1 inhibition on cancer growth is mediated through activation of apoptotic process. However, we could not observe the expression of pro-apoptotic proteins such as cleaved form Caspase 3 or Bax by Olaparib treatment, which may suggest that the Olaparib effect is not likely to be mediated by apoptotic process (Figure 2E).

On the other hand, FOXO3A has been known to trigger DNA repair in response to DNA damage by activating cell cycle arrest [28–30]. With this concern, we evaluated the effect of Olaparib in cell cycle system by performing flow cytometry analysis. Treatment of Olaparib (10 μM) significantly increased the percentage of G2/M phase cells (21%, 23%, and 28%, at day 1, 2, and 3, respectively) compared to those of control cells (11%, 15%, and 19% at day 1, 2, and 3, respectively) (Figure 2F). In addition, when FOXO3A expression was knocked down by siRNAs, the percentage of cells with G2/M arrest by Olaparib was significantly decreased from 45.8% to 29.8% (Figure 2G). These results consistently suggest that PARP1 inhibition can induce G2/M cell cycle arrest through activation of FOXO3A in gastric cancer cells.

Previously, impaired BRCA1/2 genes have been known to play an important role in conferring sensitivity to PARP1 inhibitors, providing a well-accepted DNA repair mechanism for the tumor-suppressive effect of PARP1 inhibitors [3, 7, 31, 32]. Indeed, we have also observed that knock-down of BRCA1 or BRCA2 could sensitize Olaparib effect in MKN28 gastric cancer cells (Supplementary Figure S1). This may imply that the Olaparib treatment might be beneficial especially to the BRCA-impaired gastric cancer patients, although further studies might be required to delineate the functional roles of BRCA genes to PARP1 and FOXO3A signaling.

### PARP1 and FOXO3A expressions are associated with clinical outcomes of gastric cancer patients

**Figure 3:**
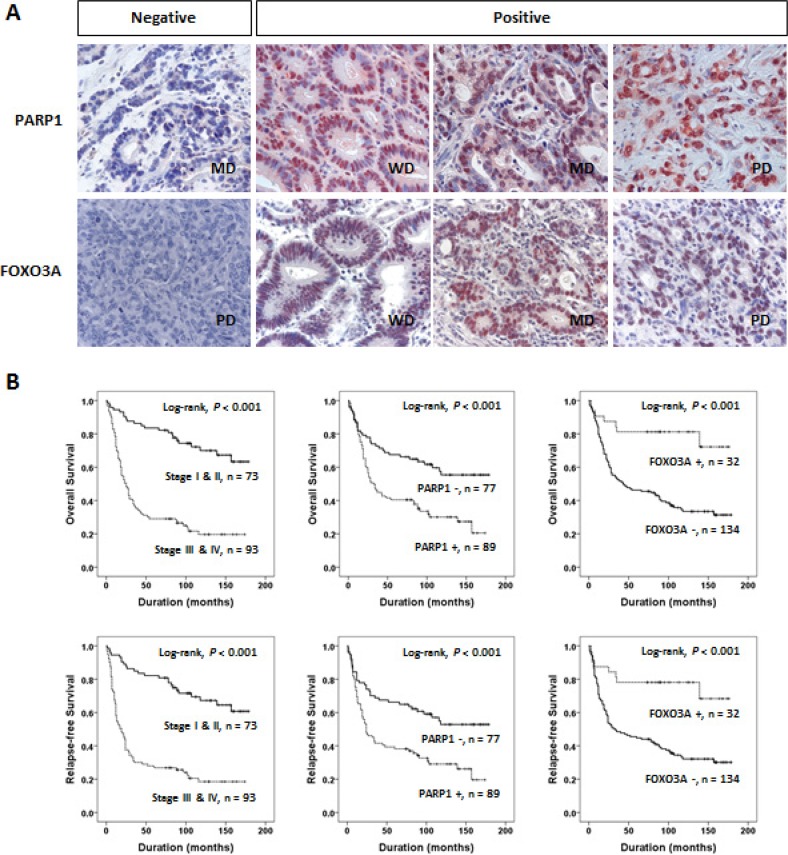
PARP1 and FOXO3A expression are associated with clinical outcomes of gastric cancer (**A**) Immunohistochemical expression of PARP1 and FOXO3A in well differentiated (WD), moderately differentiated (MD), and poorly differentiated (PD) gastric adenocarcinomas. (**B**) Kaplan-Meier plot analyses of OS (*top*) and RFS (*bottom*) for the subgroups classified based on the tumor stage (stage I and II *vs.* III and IV) and the expression status of PARP1 and FOXO3A, respectively.

Confirming the biological relevance of the PARP1 and FOXO3A expression in gastric cancer cells, we next evaluated the clinical significance of these genes by performing tissue microarray assays in the 166 cases of gastric cancer patients. Immunohistochemical staining indicated that the expressions of PARP1 and FOXO3A were mainly localized to the nuclei of tumor cells with weak expression in the cytoplasm, therefore, we considered only the nuclear expression of PARP1 and FOXO3A in the analysis (Figure 3A). By performing receiver operating characteristic curve (ROC) analysis for patients' survival, the cutoffs for positive immunostaining of PARP1 and FOXO3A were determined to have the highest likelihood ratio by selecting the cutoff values at the highest and the lowest AUC (area under the curve), respectively. Based on this analysis, PARP1-positive group was determined with cutoff score 7, and FOXO3A-positive group was determined with cutoff score 6 (Supplementary Figure S2).

Clinico-pathological features and the expression status of PARP1 and FOXO3A in the cohort of gastric cancer patients were summarized in Table 1. Overall, positive staining of PARP1 and FOXO3A proteins were observed in 54% (89 out of 166) and 19% (32 out of 166) in the cohort, respectively. PARP1 expression was more frequent in the patients with higher tumor stage (*P* < 0.001), the presence of tumor invasion (*P* < 0.001), lymph node metastasis (*P* < 0.001), and venous invasion (*P =* 0.017). In contrast, FOXO3A expression was more frequent in the patients with low tumor stage (*P =* 0.019) and early gastric cancers (EGC) (*P =* 0.042). Thus, we could suggest that PARP1 expression is associated with aggressive phenotypes while FOXO3A expression is associated with less aggressive phenotypes of gastric cancers.

**Table 1:** Clinico-pathological features and the expression status of PARP1 and FOXO3A in gastric cancer patients (*n* = 166)

Characteristic		*n*	PARP1		FOXO3A	
			Positive	*P*-value	Positive	*P*-value
Age (years)	< 60 y	50	21 (42%)	0.049	6 (12%)	0.119
	≥ 60 y	116	68 (59%)		26 (22%)	
Sex	Female	41	24 (59%)	0.466	7 (17%)	0.68
	Male	125	65 (52%)		25 (20%)	
CEA*	Normal	106	52 (49%)	0.088	18 (17%)	0.428
	Elevated	30	20 (67%)		7 (23%)	
CA19-9*	Normal	120	63 (53%)	0.778	24 (20%)	0.182
	Elevated	16	9 (56%)		1 (6%)	
TNM stage	I & II	73	28 (38%)	< 0.001	20 (27%)	0.019
	III & IV	93	61 (66%)		12 (13%)	
Tumor invasion	EGC	31	8 (26%)	< 0.001	10 (32%)	0.042
	AGC	135	81 (60%)		22 (16%)	
LN metastasis	Absence	56	18 (32%)	< 0.001	14 (25%)	0.182
	Presence	110	71 (65%)		18 (16%)	
Venous invasion	Absence	136	67 (49%)	0.017	27 (20%)	0.689
	Presence	30	22 (73%)		5 (17%)	
WHO classification	Tubular	115	70 (61%)	0.005	28 (24%)	0.045
	SRC	18	5 (28%)		1 (6%)	
	Mucinous	17	4 (24%)		1 (6%)	
	Mixed	12	6 (50%)		0 (0%)	
	Papillary	2	2 (100%)		1 (50%)	
	Neuroendocrine	2	2 (100%)		1 (50%)	
Histologic grade**	WD	10	5 (50%)	0.207	4 (40%)	0.193
	MD	64	44 (69%)		18 (28%)	
	PD	43	23 (53%)		7 (16%)	
Lauren classification	Intestinal	73	43 (59%)	0.035	20 (27%)	0.007
	Diffuse	72	31 (43%)		6 (8%)	
	Mixed	21	15 (71%)		6 (29%)	
FOXO3A	Positive	32	23 (72%)	0.021		
	Negative	134	66 (49%)			

Abbreviations: CEA, carcinoembryonic antigen; LN, lymph node; WD, well differentiated; MD, moderately differentiated; PD, poorly differentiated; SRC, signet ring cell carcinoma; EGC, early gastric cancer; AGC, advanced gastric cancer;

* Preoperative serum level of CEA or CA19-9 were not measured in 30 patients, respectively.

** Histologic grade was applied primarily to tubular and papillary carcinomas according to the WHO histological classification of gastric tumors.

Univariate Cox regression analysis has revealed several clinical features associated with shorter overall survival (OS) and/or recurrence-free survival (RFS), which included the preoperative serum levels of CEA and CA 19–9, tumor stage, lymph node metastasis, tumor invasion classification, and venous invasion (Table 2). In addition, the patient group with PARP1-positive tumors showed significant shorter OS (Hazard Ratio *HR*; 2.19, 95% CI; 1.424–3.370, *P* < 0.001) and RFS (*HR*; 2.143, 95% CI; 1.406–3.265, *P* < 0.001). Vice versa, the patients with FOXO3A-negative tumors showed shorter OS (*HR*; 3.893, 95% CI; 1.800–8.416, *P* < 0.001) and RFS (*HR*; 3.453, 95% CI; 1.673–7.127, *P* < 0.001). Kaplan-Meier analyses also revealed that these features (*i.e.*, tumor stage, PARP1, and FOXO3A) could predict prognostic outcomes of OS and RFS, respectively (log-rank test, *P* < 0.001, Figure 3B).

**Table 2:** Univariate and multivariate analysis for relapse-free survival and overall survival in gastric carcinoma patients

Features	*n*	OS		RFS	
		HR (95% CI)	*P*-value	HR (95% CI)	*P*-value
**Univariate analysis**					
CEA, elevated (*vs.* normal)	30/136	2.024 (1.230–3.330)	0.006	1.917 (1.170–3.144)	0.01
CA19-9, elevated (*vs.* normal)	16/136	2.510 (1.376–4.580)	0.003	2.275 (1.251–4.138)	0.007
TNM stage, III and IV (*vs.* I and II)	93/166	4.660 (2.865–7.581)	< 0.001	4.488 (2.802–7.186)	< 0.001
Tumor invasion, AGC (*vs.* EGC)	135/166	3.539 (1.711–7.320)	< 0.001	3.767 (1.823–7.785)	< 0.001
LN metastasis, presence (*vs.* absence)	110/166	3.696 (2.178–6.271)	< 0.001	3.737 (2.230–6.261)	< 0.001
Venous invasion, presence (*vs.* absence)	30/166	2.724 (1.705–4.352)	< 0.001	2.654 (1.666–4.229)	< 0.001
PARP1, positive (*vs.* negative)	89/166	2.190 (1.424–3.370)	< 0.001	2.143 (1.406–3.265)	< 0.001
FOXO3A, negative (*vs.* positive)	134/166	3.893 (1.800–8.416)	< 0.001	3.453 (1.673-7.127)	< 0.001
Multivariate analysis*					
TNM stage, III and IV (*vs.* I and II)		3.444 (1.922–6.171)	< 0.001	3.345 (1.918–5.836)	< 0.001
PARP1, positive (*vs.* negative)		1.783 (1.090–2.915)	0.021	1.756 (1.089–2.830)	0.021
FOXO3A, negative (*vs.* positive)		6.958 (2.163–22.383)	0.001	5.351 (1.929–14.845)	0.001

Abbreviations: OS, overall survival;RFS, relapse-free survival; HR, hazard ratio; CEA, carcinoembryonic antigen;LN, lymph node; EGC, early gastric cancer; AGC, advanced gastric cancer.

* Thevariables considered in the multivariate analysis were the pretreatment serumlevel of CEA and CA19-9, tumor stage, tumor invasion (EGC *versus* AGC),the presence of lymph node metastasis, venous invasion, and the expression ofPARP1 and FOXO3A.

In addition, to verify whether the prognostic significance of the PARP1 and FOXO3A expressions are independent each other, we performed Kaplan-Meier plot analyses for the subgroups classified based on the expression status of PARP1 or FOXO3A, respectively. We could observe that the expression status of PARP1 predicted prognostic subgroups regardless of their expression status of FOXO3A (Supplementary Figure S3A, S3B). Likewise, the expression status of FOXO3A could predict prognostic subgroups regardless of their PARP1 expression status (Supplementary Figure S3C, S3D). Consistently, multivariate analysis also demonstrated that tumor stage and the expressions PARP1 and FOXO3A are independent prognostic indicators for OS and RFS (Table 2). Therefore, we could suggest that the PARP1 and FOXO3A expressions are significantly helpful in predicting clinical outcomes of gastric cancer patients.

In Table 1, we have observed that the expressions of PARP1 and FOXO3A proteins were significantly associated with Lauren classification (*P* = 0.035 and *P* = 0.007, respectively). This may raise a possibility that the Olaparib effect and/or the prognostic association of PARP1 and FOXO3A might be different among the subtypes of Lauren classification. To address this issue, Kaplan-Meier plot analyses were performed on the each of subtypes of Lauren classification. Regardless of the subtype, we could observe that PARP1-positive tumors have poorer OS and RFS, while the FOXO3A-positive tumors have favorable OS and RFS, respectively (Supplementary Figure S4). In addition, we examined whether PARP1 inhibition has different effects among the tumor types. Treatment of Olaparib suppressed the growth of both diffuse type-derived gastric cancer cells (*i.e.*, MKN45 and KATOIII) as well as intestinal type-derived cells (*i.e.*, MKN28 and MKN74), suggesting that Olaparib is effective to suppress gastric cancer growth independent of the subtypes of Lauren classification (Supplementary Figure S5).

**Figure 4:**
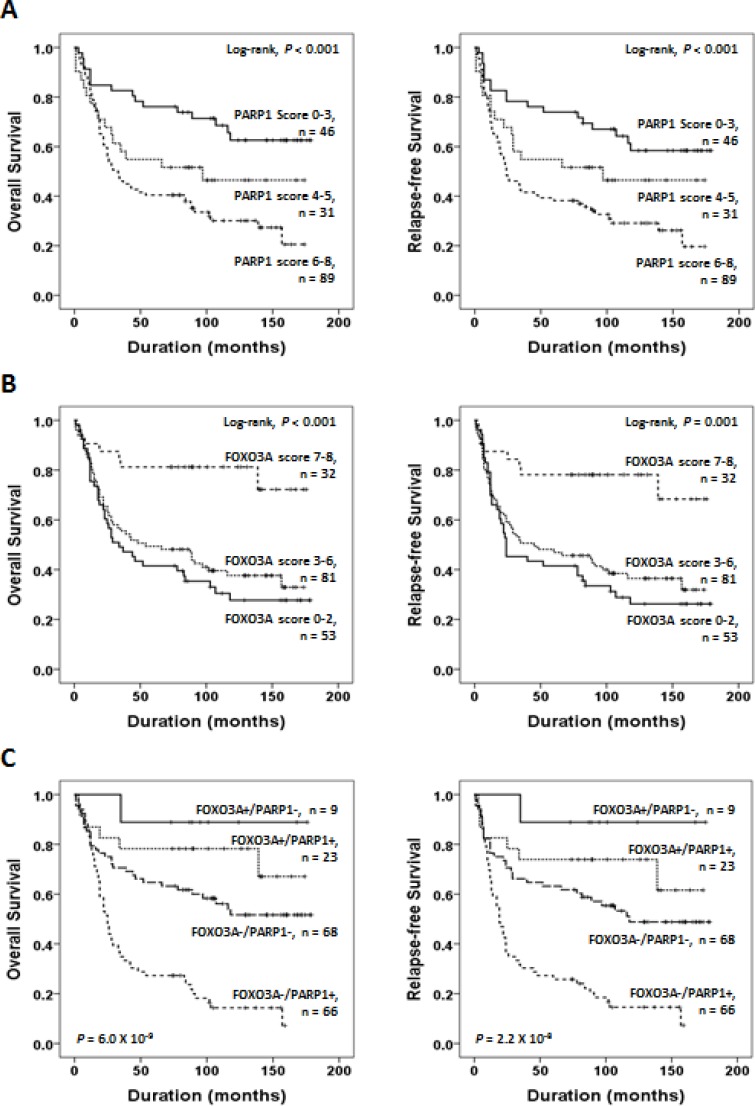
Survival analyses of PARP1 and FOXO3A expression status (**A**) Kaplan-Meier plot analyses of OS (*left*) and RFS (*right*) for the subgroups classified based on the Allen scores of PARP1 (scores 0–3, 4–5, 6–8) expression. (**B**) Kaplan-Meier plot analyses for OS (*left*) and RFS (*right*) on the subgroups classified based on the Allred scores of FOXO3A (scores 0–2, 3–6, 7–8) expression. (**C**) Kaplan-Meier survival analyses of OS (*left*) and RFS (*right*) for the subgroups classified based on the combined expression status of PARP1 and FOXO3A, *i.e.*, FOXO3A+/PARP1+ (*n* = 23), FOXO3A+/PARP1− (*n* = 9), FOXO3A-/PARP1+ (*n* = 66), FOXO3A−/PARP1− (*n* = 68). The *p*-values presented in (C) indicate the statistical significance of the log-rank test for survival among the 4 subgroups.

Next, to further verify the reliability and significance of the prognostic values of PARP1 and FOXO3A expression, we evaluated whether the intensity scores of PARP1 or FOXO3A expression are correlated with clinical outcomes. We measured the intensity of immunohistochemical staining by Allred scores for PARP1 (Mean Allred score ± standard error; 4.8 ± 0.2) and FOXO3A (Mean Allred score ± standard error; 3.8 ± 0.2). The patients were sub-classified based on the Allred scores for immunostaining intensities of PARP1 (scores 0–3, 4–5, 6–8) or FOXO3A (scores 0–2, 3–6, 7–8), respectively. We found that the patient group with higher scores of PARP1 showed poorer prognostic outcomes of OS and RFS (*P* < 0.001, Figure 4A). Vice versa, the patient group with higher scores of FOXO3A showed more favorable clinical outcomes of OS and RFS (*P* < 0.001, Figure 4B).

On the other hand, we could observe that the PARP1-positive tumors frequently co-expressed FOXO3A protein (23 out of 32, *P =* 0.021, Chi-square test, Table 1). Likewise, the Allred scores of PARP1 and FOXO3A showed significant positive correlation (Spearman's rho 0.382, *P* < 0.001, Supplementary Figure S6), implying functional link between PARP1 and FOXO3A expression. However, as shown in Table 1, PARP1-positive tumors showed aggressive phenotype, while the FOXO3A-positive tumors showed favorable phenotype. This discrepant observation raises a possibility that the oncogenic expression of PARP1 may lead to concomitant expression of FOXO3A as a compensatory mechanism to suppress tumor progression. The tumors expressing PARP1 without counter-balanced FOXO3A expression (*i.e.*, FOXO3A−/PARP1+) might acquire uncontrolled tumor growth provoking aggressive behavior.

Considering the functional link between PARP1 and FOXO3A, we next sought whether the combined expression status of PARP1 and FOXO3A is better in predicting clinical outcomes of gastric cancer patients. Strikingly, when we stratified the patients based on the combined expression status of PARP1 and FOXO3A, the patient group with PARP1+/FOXO3A− (*n* = 66) showed the worst prognosis while the patients with PARP−/FOXO3A+ (*n* = 9) showed the most favorable prognosis of OS (*P* = 6.0 × 10^−9^) and RFS (*P* = 2.2 × 10^−8^), respectively (Figure 4C). The patent group with PARP+/FOXO3A+ (*n* = 23) or the group with PARP1−/FOXO3A− (*n* = 23) showed intermediate prognostic outcomes. These results strongly suggest that the combined expression status of PARP1 and FOXO3A rather than the expression of each gene is better in predicting clinical outcomes of gastric cancer patients.

## DISCUSSION

In the present study, by performing combined analysis of experimental and clinical data, we could demonstrate that PARP1 and FOXO3A are functionally linked and their expression levels are useful in predicting clinical outcomes of gastric cancer patients. We demonstrated that PARP1 inhibition induce G2/M cell cycle arrest but not apoptosis in gastric cancer cells. In addition, FOXO3A was suggested as a downstream target for the tumor-suppressive effect of PARP1 inhibitor.

Indeed, there are a few reports that suggest a possible link between PARP1 and FOXO3A. PARP1 is activated within a few seconds after DNA damage to interact with and PARsylate (add poly ADP ribose) IKK gamma, and which promote cancer cell survival via NF- κB activation [33]. PARP1 is recruited to DNA damage sites immediately and forms interactions with a variety of nuclear DNA repair-related proteins such as ATM and PIASy [34]. This suggests that PARP1 inhibition can inhibit NF-κB /IKK-mediated pro-survival signaling in cancer cells. On the other hand, it has been suggested that ubiquitination by IKK beta can activate FOXO3A *in vitro* and *in vivo* [35, 36]. Moreover, PARP1 inhibitors could attenuate AKT phosphorylation via up-regulation of PHLPP1 [37]. AKT is known to phosphorylate and degrade FOXO3 proteins [19, 20]. These results imply that PARP1 can regulate FOXO3A indirectly through NF-κB or AKT pathways, playing important roles in tumor progression. Supporting this hypothesis, our result successfully demonstrated that FOXO3A is a possible downstream target of PARP1, although further studies might be required to delineate the precise molecular mechanisms on the PARP1 mediated regulation of FOXO3A expression.

In addition, we have addressed clinical utilities of PARP1 and FOXO3A expression for gastric cancer patients. Survival analyses using tissue microarrays have successfully demonstrated that the expression of PARP1 and FOXO3A are independent prognostic indicators (OS and RFS) for gastric cancer patients. We have also observed that PARP1 is frequently co-expressed with FOXO3A. According to this discrepant result, we hypothesized that PARP1 expression by oncogenic stimuli may induce FOXO3A expression to attenuate cancer progression as a negative feedback. Thus, if there is a defect in the compensatory expression of FOXO3A, tumors may progress to harbor uncontrolled aggressive behaviors. This raises an idea that induction of FOXO3A in cancer cells could be a new therapeutic strategy for gastric cancer treatment.

In conclusion, our results from cell culture experiments and clinical data analysis consistently indicate that the expression of PARP1 and FOXO3A play pivotal roles in gastric cancer progression. Undoubtedly, PARP1 and FOXO3A can be new prognostic and therapeutic targets for gastric cancer management.

## MATERIALS AND METHODS

### Cell culture

Human gastric carcinoma cell lines (MKN28, MKN74, NCIN87, MKN45, and KATOIII) obtained from the American Type Culture Collection (Manassas, VA) were maintained in RPMI 1640 media supplemented with 10% FBS (HyClone, Rogan, UT) and 5% penicillin/streptomycin at 37°C in a humidified 5% CO_2_ incubator.

### Chemical reagents

Mouse anti-β-actin antibody and dimethyl sulfoxide (DMSO), glycerol, glycine, sodium chloride, Thiazolyl Blue Tetrazolium Bromide, Trizma base, and Tween20 were purchased from Sigma (St. Louis, MO). Mouse anti-PARP1 and rabbit anti-FOXO3A antibodies were purchased from Santa Cruz (Santa Cruz, CA). Rabbit anti-caspase3, and rabbit anti-Bax antibody were purchased from Cell Signaling (Danvers, MA). Mouse anti-BRCA1 and rabbit anti-BRCA2 antibodies were from Abcam (Cambridge, MA). Goat anti-mouse and goat anti-rabbit horseradish peroxidase-conjugated IgG were obtained from Jackson ImmunoResearch (West Grove, PA). ECL Western Blotting Detection Reagents were obtained from Genedepot (Barker, TX). Olaparib was obtained from Selleckchem.

### siRNA mediated knock-down

Non-targeting control siRNA and siRNAs against human PARP1, FOXO3A, BRCA1 and BRCA2 were purchased from Santa Cruz Biotechnology (Santa Cruz, CA). Cells were transfected with each siRNA using Lipofectamine 2000 (Invitrogen, CA) according to the manufacturer's instruction.

### Colony formation assay

Cells (0.5 × 10^3^) were seeded in 6 cm dishes and incubated at 37°C in a humidified incubator containing 5% CO_2_ for 18 h. After incubation, cells were treated with DMSO as a control vehicle and the indicated concentration of Olaparib (2.5 μM) or PARP1 siRNA (100 nM) for 14 days. Then, the colonies were washed 2 times with PBS, fixed with 3.7% Paraformaldehyde, and stained with 1% crystal violet solution in distilled water.

### Cell counting assay

Cells (1 × 10^4^) were seeded in 6 cm dishes and incubated at 37°C in a humidified incubator containing 5% CO_2_ for 18 hr. After incubation, cells were treated with DMSO as a control vehicle and the indicated concentration of Olaparib (2.5 μM) for 0, 24, 72, and 90 h. At each time point, cell numbers were counted by using a hemocytometer.

### Real-time qPCR

Total RNA was prepared from cells using the mirVana^TM^ miRNA Isolation kit (Ambion). cDNA synthesis was performed from 2 μg of total RNA via the High Capacity cDNA Reverse Transcription kit (Applied Biosystem). The levels of gene expression were determined using a CF96^TM^ Optics Module (BIO-RAD) with the following primers: 5′-CTA CTC GGT CCA AGA TCG-3′ (sense for PARP1); 5′-TTG AAA AAG CCC TAA AGG CTC A-3′ (antisense for PARP1); 5′-TCT ACG AGT GGA TGG TGC GTT-3′ (sense for FOXO3A); 5′-CGA CTA TGC AGT GAC AGG TTG TG-3′ (antisense for FOXO3A); 5′-ACC CAG AAG ACT GTG GAT GG-3′ (sense for GAPDH); 5′-TTC TAG ACG GCA GGT CAG GT-3′ (antisense for GAPDH). All reactions were duplicated, and the 2^−ΔΔCt^ calculation was used for quantification.

### Western blotting

Cells were washed twice with phosphate-buffered saline (PBS) and lysed with lysis buffer containing protease and phosphatase inhibitors at 4°C for 30 min. After centrifuging the lysates at 10,000 rpm for 10 min, equivalent amounts of each protein lysates were resolved by 6%, 10%, or 12% SDS-polyacrylamide gel electrophoresis (PAGE) and transferred onto nitrocellulose membranes (Bio-Rad). Membranes were blocked for 1 h in 3% bovine serum albumin (BSA) in Tris buffered saline containing 0.1% Tween 20 (TBST) and then incubated for 1 h at room temperature or at 4°C overnight with primary antibody diluted in TBST containing 3% BSA. After three washes with TBST, membranes were incubated for 1 h with horseradish peroxidase-conjugated secondary antibodies (1:3000 or 1:5000 dilutions) in TBST containing 1% BSA. The immunoblots were visualized by an enhanced chemiluminescence kit obtained from West-Q ECL Platinum Solution obtained from GenDEPOT (Barker, TX).

### MTT assay

Cells (2 × 10^3^) were seeded in 96-well plates and incubated at 37°C in a humidified incubator containing 5% CO_2_ overnight. After treatment of reagents for indicated time period, 20 μL MTT solution (5 mg/mL in phosphate buffer) was added to each well and incubated for 2 h. The blue crystalline precipitate in each well was dissolved in DMSO (200 μL), and the visible absorbance at 595 nm of each well was quantified using a microplate reader.

### Flow cytometry analysis

After treatment with the Olaparib or FOXO3A siRNA, cells were harvested and washed twice with PBS and fixed in 70% cold ethanol at 4°C overnight. Before analysis, cells were washed twice with PBS, then re-suspended with 400 μL PBS and treated with 100 μg/mL RNase A (Sigma-Aldrich) and 50 μg/mL propidium iodide (PI) (Sigma-Aldrich). After incubation for 30 min at 37°C, the cells were subjected to DNA content analysis. PI fluorescence was analyzed with FACS CantoII (Becton Dickinson). Data from at least 10,000 cells were analyzed with BD FACSDIVA 7.0 (Becton Dickinson). Cell cycle distribution was calculated with ModiFit LT software.

### Patients and specimens

Tissue microarray analysis was performed using gastric adenocarcinoma specimens, which were used in a previous study [38]. In this study, we included 166 cases out of 177 cases of the previous gastric cancer specimens, because the 11 cases were unsuitable for establishing new tissue-microarrays. The patients underwent radical gastrectomy between January 1997 and December 2005 at Chonbuk National University Hospital. The patients' tumor stages (originally 50 cases for the each stage I, II, III, and IV) were matched for the gender, age (± 2 years), and calendar year of surgery- (± 2 years) based on the 6th edition of AJCC (American Joint Committee on Cancer) staging system. Thereafter, we re-staged the 166 cases according to the guidelines of the 7th edition of the AJCC staging system and reviewed them according to the criteria of the World Health Organization (WHO) classification. This study was approved by Chonbuk National University Hospital's institutional review board. Informed consent was provided according to the Declaration of Helsinki. The patients were grouped according to their age, sex, preoperative serum carcinoembryonic antigen (CEA) and CA 19–9 levels, tumor stage (I and II *vs.* III and IV), presence of lymph node metastasis, presence of distant metastasis, presence of venous invasion, histologic types according to the WHO classification, histologic grade of tubular and papillary type carcinomas, Lauren classifications, and tumor invasion [early gastric carcinoma (EGC) *vs.* advanced gastric carcinoma (AGC)]. The follow-up end point for patients survival and recurrence-free survival was the date of the last contact or the date of death through December 2011.

### Tissue microarrays

Immunohistochemistry was performed on paraffin-embedded tissue-microarray blocks. One 3.0 mm core per case was analyzed in a tissue-microarray. The tissue sections were treated with a microwave antigen retrieval procedure in pH 6.0 sodium citrate buffer for 20 minutes. The following markers were used: PARP1 (H-300) (1:100, Santa Cruz Biotechnology, Santa Cruz, CA), and FOXO3A (D19A7) (1:100, Cell Signaling Technology, Beverly, MA). Two pathologists (Jang KY and Kim KM) have evaluated the immunohistochemical staining of PARP1 and FOXO3A in tissue microarrays by consensus without knowledge of the clinicopathological information. The scoring for the immunostaining for PARP1 and FOXO3A was performed according to the Allred scoring system, which is generally used for evaluation of nuclear expression [39, 40]. The nuclear staining intensity was scored as 0 (no staining), 1 (weak staining), 2 (intermediate staining), or 3 (strong staining). The area of staining was evaluated using the following scores: 0, no staining cells; 1, 1% of the cells stained positive; 2, 2–10% of the cells stained positive; 3, 11–33% of the cells stained positive; 4, 34–66% of the cells stained positive; and 5, 66–100% of the cells stained positive. Thereafter, the sum of intensity score and proportion score was used for further analysis. The maximum sum score was 8 and the minimum sum score was zero.

### Statistical analysis

Significance of associations between variable clinicopathologic factors and the expressions of PARP1 or FOXO3A were estimated by Pearson's chi-square test. Univariate and multivariate Cox proportional hazards regression analyses and Kaplan-Meier survival analysis were performed using SPSS software (version 19.0).

## SUPPLEMENTARY MATERIAL FIGURES


