# Supplementary material for: Tumor suppressive effect of PARP1 and FOXO3A in gastric cancers and its clinical implications
Source: Oncotarget. 2015 Nov 2;6(42):44819–31. doi: 10.18632/oncotarget.6264 (PMC4792594; doi:10.18632/oncotarget.6264)
Supplement: Supplementary file 1 [file oncotarget-06-44819-s001.pdf]

Tumor suppressive effect of PARP1 and FOXO3A in gastric cancers and its clinical implications

Supplementary Materials

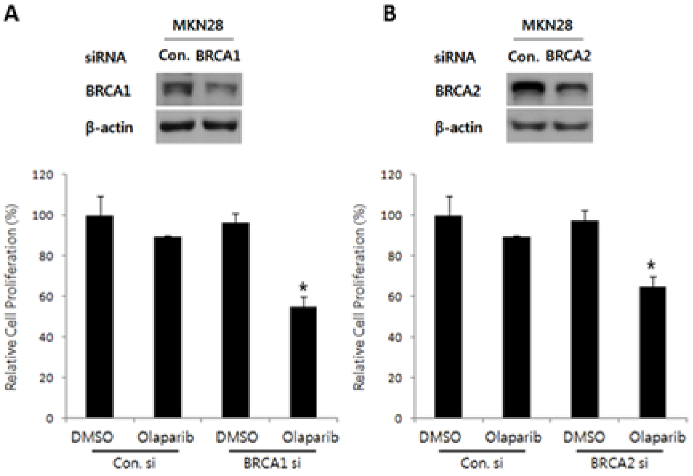

Supplementary Figure S1: Effect of BRCA1/2 knock-down on the Olaparib-mediated inhibition of cancer cell growth. (**A, B**) Olaparib (0 and 2.5 µM, for 72 h) is treated against the MKN28 cells transfected with control or BRCA1 (A) or BRCA2 (B) siRNA, and the cell proliferation is determined by MTT assay. *$p < 0.001$. Error bars indicate the standard deviation of triple replicates.

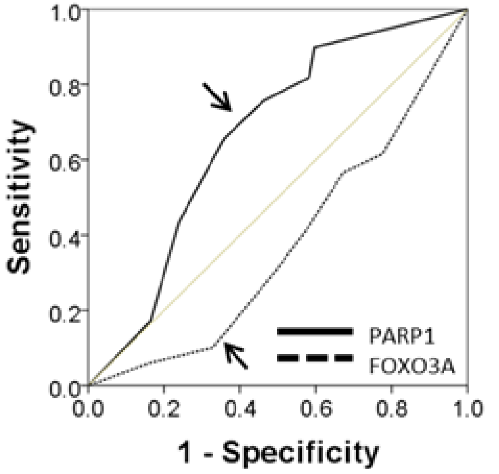

	Cut-Off	AUC	*P-value*
PARP1	All	0.665	< 0.001
	≥ 7	0.649	0.001
FOXO3A	All	0.371	0.005
	≥ 6	0.386	0.013

Supplementary Figure S2: Statistical analysis to determine cutoff values for the immunohistochemical staining PARP1 and FOXO3A. The cut-off values are determined by the receiver operating characteristic curve (ROC) analysis for the estimation of overall survival of patients. The cutoff values for the PARP1 and FOXO3A expression are determined at the highest AUC (area under the curve) and the lowest AUC values, respectively. Arrow indicates the determined cutoff point in ROC for PARP1 and FOXO3A (*top*). The cutoff for PARP1 expression is determined to score 7, and the cutoff for FOXO3A expression is determined to score 6 (*bottom*).

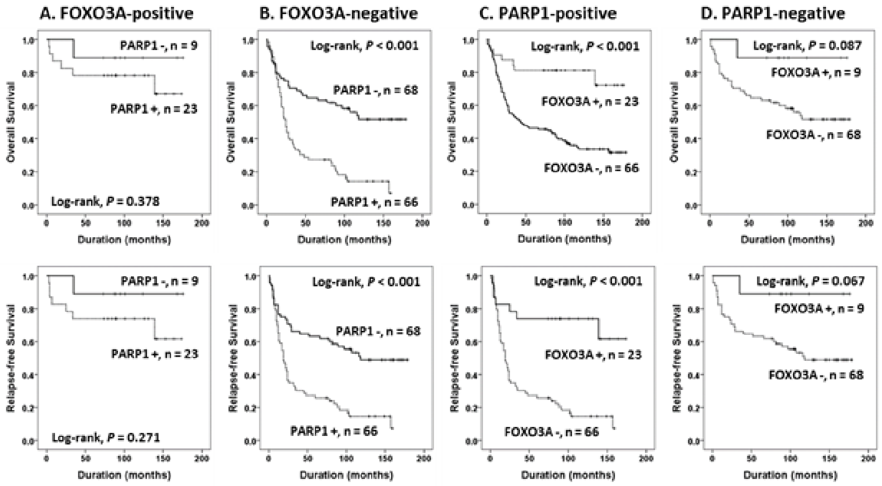

Supplementary Figure S3: Kaplan-Meier survival analyses for the expression status of PARP1 and FOXO3A in the subgroups based on the expression of PARP1 or FOXO3A. (**A, B**) Kaplan-Meier plot analyses for overall survival (OS, *top*) and relapse-free survival (RFS, *bottom*) according to the expression status of PARP1 in FOXO3A-positive (A) and FOXO3A-negative (B) subgroups. (**C, D**) Kaplan-Meier plot analyses for overall survival (OS) and relapse-free survival (RFS) according to the expression status of FOXO3A in PARP1-positive (C) and PARP1-negative (D) subgroups.

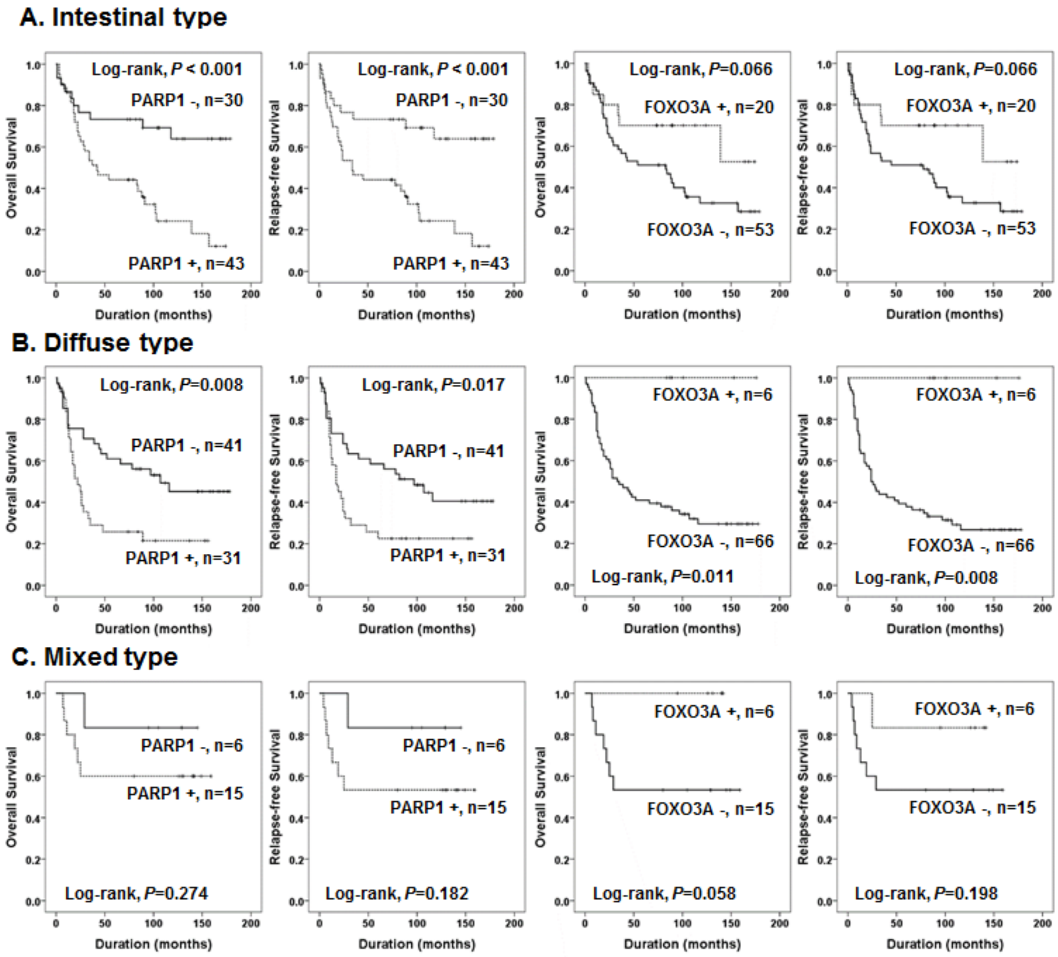

Supplementary Figure S4: Kaplan-Meier survival analyses for the expression status of PARP1 and FOXO3A in the subgroups of Lauren Classification. Kaplan-Meier plot analyses for overall survival (OS) and relapse-free survival (RFS) according to the expression status of PARP1 or FOXO3A in each subgroup of intestinal (**A**) diffuse (**B**) and mixed (**C**) type of Lauren Classification.

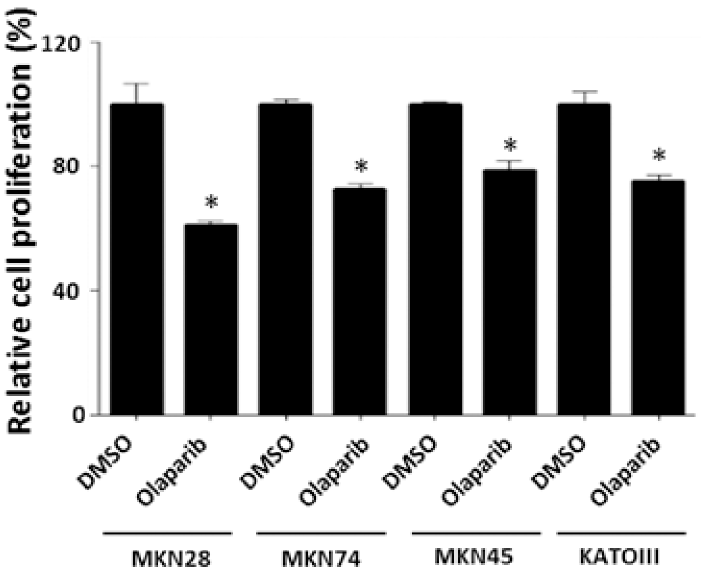

Supplementary Figure S5: Olaparib effect on the cell growth of intestinal type (MKN28, MKN74) and diffuse type (MKN45, KATOIII)-derived gastric cancer cells. Cell proliferation rates are measured by MTT assay on the MKN28, MKN74, MKN45 and KATOIII cells with presence (10 μM) or absence (DMSO vehicle) of Olaparib for 72 h, respectively. Error bars indicate standard deviation for triple replicates. *$P < 0.05$ with respective control.

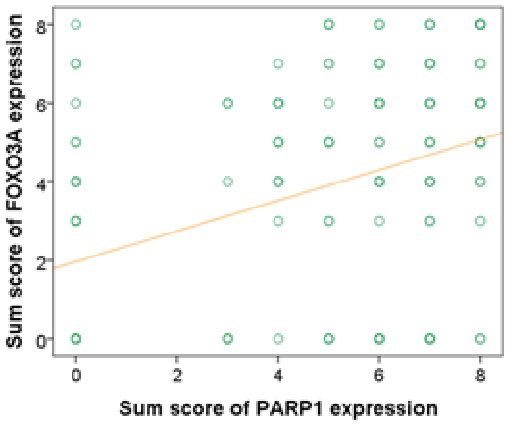

Supplementary Figure S6: Correlation of PARP1 and FOXO3A immunostaining scores. A scatter plot shows positive correlation between the immunohistochemical Allred scores of PARP1 and FOXO3A in the 166 cases of tissue microarray data of gastric cancer patients ($R^2 = 0.147$, Spearman's rho 0.382, $P < 0.001$).
